# Systemic therapy for vulval Erosive Lichen Planus (the ‘hELP’ trial): study protocol for a randomised controlled trial

**DOI:** 10.1186/s13063-015-1133-z

**Published:** 2016-01-04

**Authors:** Rosalind C. Simpson, Ruth Murphy, Daniel J. Bratton, Matthew R. Sydes, Sally Wilkes, Helen Nankervis, Shelley Dowey, Kim S. Thomas

**Affiliations:** Centre of Evidence Based Dermatology, University of Nottingham, King’s Meadow Campus, Lenton Lane, NG7 2NR Nottingham, UK; Nottingham University Hospitals, Queen’s Medical Centre Campus, Derby Road, Nottingham, NG7 2UH UK; MRC Clinical Trials Unit at UCL, Institute of Clinical Trials and Methodology, Aviation House, 125, Kingsway, London, WC2B 6NH UK

**Keywords:** Multi-armed, Randomised controlled trial, Pragmatic, Open-label, Pilot, Dermatology, Vulval, Erosive lichen planus, Therapy

## Abstract

**Background:**

Erosive lichen planus affecting the vulva (ELPV) is a relatively rare, chronic condition causing painful raw areas in the vulvovaginal region. Symptoms are pain and burning, which impact upon daily living. There is paucity of evidence regarding therapy. A 2012 Cochrane systematic review found no randomised controlled trials (RCTs) in this field. Topically administered corticosteroids are the accepted first-line therapy: however, there is uncertainty as to which second-line treatments to use. Several systemic agents have been clinically noted to show promise for ELPV refractory to topically administered corticosteroids but there is no RCT evidence to support these. The ‘hELP’ study is a RCT with an internal pilot phase designed to provide high-quality evidence.

**Methods/Design:**

The objective is to test whether systemic therapy in addition to standard topical therapy is a beneficial second-line treatment for ELPV. Adjunctive systemic therapies used are hydroxychloroquine, methotrexate, mycophenolate mofetil and prednisolone. Topical therapy plus a short course of prednisolone given orally is considered the comparator intervention. The trial is a four-armed, open-label, pragmatic RCT which uses a blinded independent clinical assessor. To provide 80 % power for each comparison, 96 participants are required in total. The pilot phase aims to recruit 40 participants.

The primary clinical outcome is the proportion of patients achieving treatment success at 6 months. ‘Success’ is defined by a composite measure of Patient Global Assessment score of 0 or 1 on a 4-point scale plus improvement from baseline on clinical photographs scored by a clinician blinded to treatment allocation. Secondary clinical outcomes include 6-month assessment of: (1) Reduction in pain/soreness; (2) Global assessment of disease; (3) Response at other affected mucosal sites; (4) Hospital Anxiety and Depression Scale scores; (5) Sexual function; (6) Health-related quality of life using ‘Short Form 36’ and ‘Skindex-29’ questionnaires; (7) Days of topical steroid use; (8) Treatment satisfaction; (9) Discontinuation of medications due to treatment failure; (10) Per participant cost of intervention in each treatment group. Adverse events will also be reported.

**Discussion:**

‘hELP’ is the first RCT to address second-line treatment of ELPV. The trial has encountered unique methodological challenges and has required collaborative efforts of the UK Dermatology Clinical Trials Network alongside expert clinicians.

**Trial registration: current controlled trials:**

ISRCTN 81883379. Date of registration 12 June 2014.

**Electronic supplementary material:**

The online version of this article (doi:10.1186/s13063-015-1133-z) contains supplementary material, which is available to authorized users.

## Background

Erosive lichen planus is a chronic inflammatory, scarring skin condition that most commonly occurs on the mucosal surfaces of the mouth and genital region. It is believed to be an autoimmune condition [1, 2] with T-cell-mediated damage to keratinocytes in the basal layer of the epidermis [3], although the exact pathogenesis remains unclear.

Erosive lichen planus affecting the vulvovaginal region (ELPV) causes painful raw areas at the vaginal entrance, and subsequent scarring leads to anatomical changes with narrowing of the vaginal canal. Symptoms can prevent normal daily activities such as walking/sitting, washing, going to the toilet and normal sexual function. Additionally, there is a 1–3 % risk of cancerous change in affected skin although the timescale for malignancy to occur is unclear [4–6].

ELPV is estimated to affect 0.01 % of women. However, this is likely an underestimate with many cases failing to present to medical services [7].

No robust, high-quality randomised controlled trials (RCTs) have been published on which to base treatment decisions for ELPV [8]. First-line therapy is typically with the super-potent topical steroid clobetasol propionate 0.05 % [9, 10]. Non-randomised studies, mainly retrospective case series, have suggested that clobetasol propionate is an appropriate first-line therapy [6, 11–13]. However, in the only prospectively-collected case series [6], one third of patients responded poorly to this type of treatment.

There is currently no agreement as to which second-line agents should be used [10, 14]. Therapeutic choice is based upon expert opinion and predominantly includes immunosuppressant or immune-modifying agents.

Expert clinicians anecdotally believe that systemic treatments with the greatest success rates are hydroxychloroquine, methotrexate, mycophenolate mofetil and prednisolone. In a UK-based multi-centre case note review of 172 patients, these were the most commonly prescribed systemic agents [9]. However, there are insufficient data within the medical literature to make an evidence-based decision on the most effective. The ‘hELP’ trial (Systemic therapy for vulval Erosive Lichen Planus) has been designed to test all four of the more common interventions in one four-armed RCT with an internal pilot.

The objectives of the ‘hELP’ study are to administer, for a 6-month period, in patients with ELPV that has been refractory to first-line therapy, clobetasol propionate 0.05 % topically plus adjuvant systemic therapy with hydroxychloroquine, methotrexate or mycophenolate mofetil, and compare against topically administered clobetasol propionate 0.05 % with adjuvant prednisolone given orally.

## Methods/Design

### Study design

The ‘hELP’ trial is a multi-centre, four-armed, open-label pragmatic RCT. The trial has a 12-month internal pilot phase. Participants are being recruited from 12 hospitals throughout the UK which hold specialist vulval clinics. The active intervention phase is for 6 months after which the primary outcome will be assessed. Participants will be followed up for 12 months after randomisation. Treatment regimens and monitoring are based upon national guidance issued by the British Association of Dermatologists [15]. Study visits mimic the schedule of clinic appointments seen in usual clinical practice, the timings of which were set following consultation with expert clinicians.

### Participants

Inclusion criteria are

i). Aged 18 or over;
ii). Clinical diagnosis of moderate to severe ELPV, despite first-line therapy for 3 months with clobetasol propionate 0.05 %;
iii). A documented vulval biopsy at some point in their history to exclude malignant/pre-malignant disease. The biopsy should be repeated if clinical features have changed or are suspicious;
iv). Willing to have clinical photographs taken;
v). If of childbearing potential, willing to use effective contraceptive methods whilst taking systemic therapy (and in the case of methotrexate for 6 months after the end of treatment);
vi). Willing to give informed consent.

Participants are ineligible if they have:

i). Lichen sclerosus/lichen planus overlap syndrome;
ii). Received one or more of the trial drugs within the last month (excluding topical therapy);
iii). A previous or current diagnosis of malignant disease;
iv). Pre-malignant cervical or vulval disease;
v). Received a live vaccine in the 2 weeks before starting trial treatment;
vi). Pregnancy or are breast-feeding;
vii). Known allergy to any of the trial medications;
viii). Previous history of clinically significant renal or liver impairment, or concurrent medications that would interact with the trial drugs;
ix). Any other reason that the trial drugs would not be given in usual clinical practice.

### Interventions

All participants receive standard topical therapy of clobetasol propionate 0.05 % once daily for 1 month and the regimen will be reduced according to response thereafter. If the condition worsens, application may be increased, but as ELPV is a relapsing remitting condition, this does not necessarily indicate treatment failure. A maximum of 60 g clobetasol propionate 0.05 % over 6 months should be used.

Participants will be randomised to receive one of four systemic treatments (in addition to topical therapy) for a period of 6 months. The 4-week course of prednisolone given orally will be considered the comparator intervention.

Topical therapy alone would have been a simpler comparator, but we considered it unethical to continue this alone as, by definition to enter the trial, topical therapy must have failed. Therefore, a short course of prednisolone given orally for the first month is given to the comparator group.

#### Intervention regimens

1. Prednisolone given orally starting at 20 mg once daily for 1 week, reducing by 5 mg per week for 4 weeks. Where clinically indicated, gastric and bone protection should be prescribed according to usual practice.
2. Oral hydroxychloroquine tablets up to 6.5 mg/kg body weight with a maximum dose of 200 mg twice daily.
3. Oral methotrexate tablets commencing at 5 mg/week and gradually titrated according to response to a maximum of 25 mg/week.
4. Oral mycophenolate mofetil tablets commencing at 500 mg once daily and gradually titrated according to response to a maximum dose of 1.5 g twice daily.

Treating clinicians are advised to titrate the dose of the active interventions, according to response and tolerability, as detailed in national guidelines issued by the British Association of Dermatologists [15]. As methotrexate is a folate antagonist it is recommended that participants are prescribed adjunctive folic acid 5 mg daily except on the day of methotrexate administration. Clinicians are allowed to use their clinical judgement to deviate from these recommendations if clinically necessary.

Participants in the RCT should not use any other topical or systemic treatments for ELPV after randomisation. There is a 4-week washout period of any pre-existing therapy with the exception of clobetasol propionate 0.05 %. All other concomitant medications should continue as per normal care. The use of live vaccines is not permitted during the intervention phase of the trial and the prescription of any additional medications for ELPV during the trial period is considered as a treatment failure.

### Outcomes

#### Feasibility outcomes

After the end of the internal pilot phase feasibility outcomes will be reported:

The feasibility primary outcome is to establish the proportion of eligible patients willing to be randomised to systemic treatment.

The feasibility secondary outcomes are:

i). To establish participants’ adherence to treatment regimens;
ii). To assess the quality of photographs obtained and suitability for blinded clinical assessment of disease severity;
iii). To assess the suitability and completion of paper-based case report forms;
iv). To trial a four-armed study design in this population and assess what implications using multiple arms has on recruitment and overall study conduct including post-randomisation dropouts;
v). To evaluate the suitability of the chosen clinical outcomes for evaluating treatment response in ELPV.

### Clinical outcomes

#### Primary clinical outcome measure

The primary outcome of the ‘hELP’ study is the *proportion of patients achieving treatment success at 6 months*. Treatment will be classed as successful if *both* patient and investigator success criteria are met:

- Patient Global Assessment of disease severity of ‘none’ or ‘mild’ (on a 4-point scale of none, mild, moderate or severe disease)
- Any improvement from baseline judged by blinded assessment of clinical photographs

The composite outcome incorporates both blinded assessment of physical signs, where blinding is important in open-label trials, and patient assessment of symptoms, which is important because physical signs do not always reflect symptoms.

#### Secondary clinical outcome measures

Secondary outcomes comprise the following:

i). Reduction in soreness from baseline at 6 months assessed using a 10-point score (0 = no soreness, 10 = most soreness);
ii). Disease severity assessed individually by: a) Patient Global Assessment at baseline, 3, 6 and 12 months, b) Investigator assessment by treating clinician at baseline, 3 and 6 months, c) Investigator assessment by blinded assessor using clinical images at baseline and 6 months;
iii). Investigator assessment of severity of oral and vaginal sites if affected at baseline, 3 and 6 months;
iv). Psychological assessment using the Hospital Anxiety and Depression Scale at baseline and 6 months;
v). Sexual function questionnaire at baseline 6 and 12 months;
vi). Health-related quality of life using ‘Skindex-29’ and ‘Short Form 36’ questionnaires at baseline and 6 months;
vii). Days of topical steroid use (as a marker of disease control in each of the groups);
viii). Overall treatment satisfaction;
ix). Economic evaluation, which will calculate the average cost of intervention in each treatment group per participant based on prescribed medication.

### Participant timelines

Informed consent will be obtained by the recruiting clinician prior to conducting any trial-specific procedures. Eligible, consented participants will have baseline symptoms and signs recorded, including a digital photograph. A thorough medical history will be taken to ensure their suitability to receive any of the trial medications. Participants will subsequently be randomised to one of the four trial arms, following which medication-specific eligibility assessments will be conducted (e.g. X-rays for the methotrexate group). Treatment will start once the results of these tests are known to be satisfactory. Standard safety monitoring will be conducted as per national guidance [15] and in accordance with the individual Summary of Medical Product Characteristics.

**Fig. 1:**
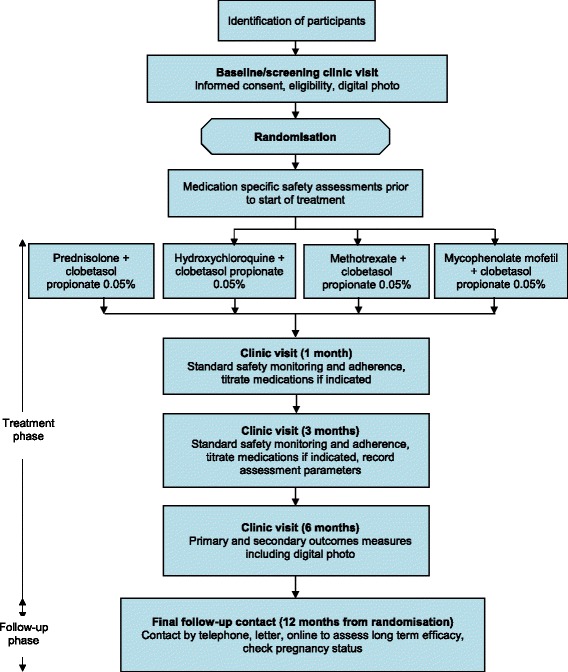
Systemic therapy for vulval Erosive Lichen Planus (‘hELP’) study flow diagram. Internal pilot phase to run for 12 months, after which recruitment rates will be assessed and a decision made whether or not to continue onto a definitive trial

Follow-up visits will be at 1, 3 and 6 months to review safety monitoring investigation results, check adherence, titrate medication dosage if required and record outcome measure parameters. Women of childbearing potential who are sexually active will have pregnancy testing carried out and the importance of effective contraception reiterated. The primary outcome is assessed after 6 months of therapy, although primary and secondary endpoint data are collected at both 3- and 6-month visits (Fig. 1).

After the 6-month visit normal care will resume; the trial medication will continue if adequate control has been achieved, otherwise an alternative approach will be chosen by the treating clinician. No further in-person trial visits will be required: however, participants will be contacted by phone or Email at 12 months by the coordinating centre to assess long-term outcomes.

### Data collection

With the exception of clinical photographs, data are collected through paper-based Case Report Forms (CRFs) and participant diaries. Assessors are trained during site initiation visits on how to complete the CRFs accurately. Clinical photographs are obtained digitally through the site’s local medical illustration department. A photographic protocol is available to ensure standardisation of image quality. Although medical illustration is the preferred method of photography, if not available at site, clinicians may take the photographs in line with the photographic protocol. It is mandatory that the same method of photography (i.e. medical illustration or clinician) is used for the two time points for an individual patient to ensure comparability of images.

Photographs and CRFs are confidential documents and are held securely. Each participant will be assigned a trial identity code number so that documented data are non-identifiable. Any personal information will be stored on a password protected, secure server and will only be looked at by authorised individuals from the University of Nottingham, the research group, and regulatory authorities where relevant.

A summary of assessments performed at the different time points is demonstrated in Table 1.

**Table 1:** Summary of the Systemic therapy for vulval Erosive Lichen Planus (‘hELP’) trial assessments to be made at study visits

Assessment	0 months			1 month	3 months	6 months	12 months^g^
	Screening/Eligibility	Baseline/Randomisation	Start of treatment				
Informed consent		✓					
Eligibility checks	✓						
Medical history	✓	✓					✓
Demographics		✓					
Randomisation		✓					
Standard safety monitoring^a^		✓		✓	✓	✓	
Pregnancy test^b^			✓	✓	✓	✓	Check status
Prescription given^c^			✓	✓	✓	✓	
Treatment log^d^			✓	✓	✓	✓	
Digital photographs^e^		✓				✓	
Pain/Soreness score^f^		✓			✓	✓	
PGA^f^		✓			✓	✓	✓
Investigator assessment of vulva		✓			✓	✓	
Investigator assessment of other affected sites		✓			✓	✓	
HADS score^f^		✓				✓	
Assessment of sexual function^f^		✓				✓	
Skindex-29^f^		✓				✓	
SF36^f^		✓				✓	
Patient diary^f^		✓		✓	✓	✓	
Adverse events				✓	✓	✓	

*HADS* Hospital Anxiety and Depression Scale, *PGA* Patient Global Assessment, *SF36* ‘Short Form 36’

^a^As per national guidance

^b^For women of childbearing potential who are sexually active

^c^Doses titrated according to ‘hELP’ study protocol

^d^Record any prescriptions given during follow-up visit

^e^Photographs ideally to be taken by medical photography (photographs at baseline and 6 months *must* be by same mode), analysis by blinded assessor at coordinating centre

^f^Assessment completed by the participant

^g^Assessment to be carried out by telephone, letter or Email

### Sample size and recruitment

Each treatment group will be compared with the comparator group on the primary outcome with adjustment for multiple testing. Assuming a comparator arm success rate (primary outcome measure) of 10 % at 6 months and using a 1:1:1:1 allocation ratio, 96 participants will be required to detect a 40 % absolute increase in the proportion of successes to 50 %, with 80 % power and maintaining the 2-sided familywise error rate at 5 % [16]. As there is very little evidence on which to base treatment effect size, feedback from clinicians and patients was that a substantial improvement would be necessary in order to justify systemic treatment to treat the condition.

The internal pilot recruitment period will be 12 months during which we aim to recruit 40 participants. If fewer than 40 participants are recruited, recruitment will stop and feasibility outcomes will be reported.

### Randomisation and blinding

Randomisation codes have been generated by computer using minimization using the Taves minimisation method [17], with an 80 % chance to minimise (VBScript within an ASP web application), and are held by the Nottingham Clinical Trials Unit. Randomisation is based on the minimisation criteria of recruiting centre and baseline disease severity. Participants who are found to be ineligible following medication-specific pre-treatment investigations will be removed from the trial and not replaced.

The randomisation sequence will be concealed until recruitment, data collection, and data cleaning are complete.

This is an assessor-blind study with part of the primary outcome measure (clinical assessment of disease improvement) being judged on the basis of anonymised and blinded digital photographs. This will protect against detection bias. Digital photographs will be assessed by two assessors who are blinded to treatment allocation. Any discrepancy will be adjudicated by a third independent assessor. The trial statistician will also be blinded until the final analysis.

Since the clinician and participants are aware of the treatment allocation, no special measures are required to allow for breaking codes. Treatment allocation will be concealed from the treating clinician until the patient’s key details and minimisation variables have been entered into the randomisation system.

### Statistical analysis

If fewer than 40 participants in 12 months are recruited, feasibility outcomes will be reported. Summary data for efficacy outcomes will still be reported in full for potential inclusion in future meta-analyses. However, significance testing and multiple imputation of missing data will not be considered.

All analyses will be performed using the intention-to-treat principle. The ‘Full Analysis Set’ will be all participants who are randomised and continue to be eligible after the post-randomisation eligibility assessments to their allocated trial arm regardless of whether they commence the study medication, or not. All primary and secondary outcome measures will be analysed using this sample of patients.

The primary clinical outcome measure will also be assessed on a per-protocol sample which includes all participants in the ‘Full Analysis Set’ who are deemed to have no major protocol violations that could interfere with the objectives of the study: for example, continuing concomitant medications that are contraindicated in conjunction with the trial treatments.

The primary clinical outcome is a composite measure comprising assessment of digital photographs and Patient Global Assessment. If digital photographs are not available for any of the participants, the assessment that has been recorded by the treating clinician as part of data collection at each trial visit will be used instead. However, if used, this assessment will be unblinded due to the open-label nature of the trial.

Multiple imputation will be used to deal with missing patient assessments or investigator assessments. Fifty imputations will be used to generate 50 complete datasets. The relevant outcome in each dataset will be analysed using the corresponding methods described above with the results across datasets combined using Rubin’s rules. Imputations will be done separately within treatment groups.

Statistical tests will be at the 2-sided 0.05 level, apart from the analysis of the primary clinical outcome measure, which will adjust for multiple comparisons using Dunnett’s step-down procedure [18] to control the familywise error rate at the 0.05 level. All analyses will be adjusted for the minimisation variables and, if applicable, the baseline measurement of the corresponding outcome.

Treatment effects for binary outcomes (including primary clinical outcome) will be analysed using a binomial regression model with identity link and will be presented as an absolute difference in proportions.

The change in continuous outcomes between baseline and follow-up will be compared between treatment arms using an absolute difference in means, estimated using a multiple linear regression model.

Categorical outcomes will be analysed using ordinal logistic regression and treatment effects will be presented using odds ratios. All models will adjust for treatment allocation and minimisation factors.

Days of topical steroid use will be analysed using a negative binomial model with the number of days of follow-up for each patients included in the model as an offset.

The chief investigator (CI) will maintain responsibility for final trial dataset and data will be available on request.

### Study organisation and funding

This study is coordinated from the Centre of Evidence Based Dermatology [19] at the University of Nottingham with support from the UK Dermatology Clinical Trials Network [20]. It is funded by the National Institute of Health Research as part of a Doctoral Research Fellowship (DRF-2012-05-166). The award holder (RS) is the trial manager. Additional trial administrative support is provided by the University of Nottingham. Research nurse support is provided through the National Institute for Health Research (NIHR) Clinical Research Networks. The research is sponsored by the University of Nottingham.

Research ethics approval has been obtained from the Sheffield NRES Committee – York and The Humber (reference 14/YH/0046).

The study protocol was developed in accordance with Standard Protocol Items: Recommendations for Interventional Trials (SPIRIT) 2013 guidelines (Additional file 1).

The CI has overall responsibility for the study and is supported by the trial’s medical expert.

The Trial Management Group (TMG), which meets monthly, includes the CI (KT), medical expert (RM), trial manager (RS), assistant trial manager (HN), clinical trials development manager (SD) and trial statistician (SW). The trial is overseen by a Trial Steering Committee (TSC), which includes an independent chair and four other independent members (one of whom is a patient with ELPV) plus representatives from the TMG. The Independent Data Monitoring Committee (IDMC) consists of three members. Meetings for the DMC and TSC occur annually or more frequently if required. All members are independent of the study team although the CI and trial manager (plus other members of the TMG as necessary) attend open sessions to inform the oversight committees of trial progress.

## Discussion

The ‘hELP’ trial has encountered some unique methodological challenges due to its pragmatic ethos and the fact that ELPV is a relatively rare condition. As the study is funded through a Doctoral Research Fellowship, investigators are contributing on a voluntary basis as they believe this is a clinically important question that will help to improve practice, and trial drugs are prescribed through normal clinical care pathways. This recruitment model has been successfully used in other UK Dermatology Clinical Trials Network Trials [21]. An example of a study previously developed in this way for a rare skin condition is the ‘STOP GAP’ trial of prednisolone versus cyclosporin for pyoderma gangrenosum [22].

The reason for designing an open-label trial is that the very different treatment regimens, and safety monitoring requirements for the four systemic therapies, meant that blinding of participants and clinicians would have been impractical and would have required each participant to additionally take multiple placebo tablets. Furthermore, it was felt that with so many different regimens and the potential side effects of some of the treatments, it could have been potentially dangerous to blind the patient and investigator to the intervention. Nevertheless, measures were taken to ensure the scientific integrity of the trial, including randomisation and allocation concealment through an accredited Clinical Trials Unit, and blinded outcome assessment using digital images to limit detection bias.

Due to the fact that the treatment groups require different pre-treatment safety assessments, the decision was made to perform treatment-specific eligibility assessments postrandomisation. It was considered unethical and impractical to perform certain tests prior to randomisation as some participants would undergo investigations (e.g. X-rays) that were not required. To minimise the potential for post-randomisation dropouts, a detailed medical history is taken and any potential medical issues investigated prior to inclusion in the study.

This is the first RCT to compare systemic therapies in ELPV, making the choice of target interventions difficult. As patient numbers are scarce and resources limited in this population, it was decided to design a four-arm study which could give a preliminary indication of which of the trial interventions is most likely to be effective.

Eligibility criteria were chosen based upon usual clinical practice. In particular, participants must have had a vulval biopsy at some point in their history. The biopsy must have excluded malignant or pre-malignant disease. The procedure is painful and distressing to patients and, therefore, the study does not require the biopsy to be repeated unless clinically suspicious features develop. Biopsy is not used to diagnose ELPV per se because classic histological features of disease are not always seen [13, 23]. In clinical practice, biopsy is used to rule out malignancy/pre-malignancy, which are in the differential diagnosis of the condition. This is particularly important if the use of immunosuppressive medications is planned. It may be considered that the need to have a documented biopsy as an inclusion criterion is a source of bias, but, it is a necessary safety precaution and would be considered negligent practice to use the trial medications without it.

The choice of interventions in the study was developed in conjunction with collaborating expert clinicians through the British Society for the Study of Vulval Disease. Although topical therapy alone as the comparator may have been the ideal choice, it was considered unethical to continue this alone as, by definition, to enter the trial topical therapy must have failed. Therefore, a short course of prednisolone given orally was added for the first month to the comparator group. As prednisolone’s effects are short-lived, disease control at 6-months for the primary outcome should be the equivalent of using topical therapy alone in this group.

As systemic therapies can take weeks or months to produce optimum effect, the rationale for using clobetasol propionate 0.05 % with the investigational adjunctive systemic therapies is that ELPV may flare severely if topical treatment is stopped abruptly. As the systemic therapies take effect, disease control should improve and patients should find that they can reduce the frequency of application of clobetasol propionate 0.05 % without experiencing flares. If the condition is not controlled by the systemic agent they can continue to use clobetasol propionate 0.05 % on a more frequent basis. Therefore, the frequency of application of clobetasol propionate 0.05 % will act as a marker of disease control achieved by the systemic agents.

With no validated outcome measures for ELPV the primary endpoint was considered at length and was devised following careful discussion with clinicians and patients. As this is an open-label trial any patient- and investigator-rated outcomes, except the independent blinded assessment of the clinical photographs, may be subject to bias. The Trial Development Group (TDG) felt it was important to maintain the integrity of the trial by going to the additional length of a blinded assessor to score clinical photographs. However, it is important that patients’ views are also taken into consideration as symptoms and impact of disease play a large part in clinicians’ therapeutic decision-making. The decision to use a composite endpoint was, therefore, made. Subjective symptoms need to be taken in context with clinical signs: therefore, improvements in both patient and investigator assessments are required to classify treatment success.

The ‘hELP’ trial is the first of its kind and, in consultation with clinicians and patients, we have endeavoured to produce a pragmatic and clinically relevant RCT within the limited resources available.

## Trial status

Recruitment into this trial started in July 2014 and is taking place in 12 secondary care hospitals in the United Kingdom. Recruitment ended on 31 July 2015.

## Additional files

Additional file 1:
**SPIRIT checklist_’hELP’ trial protocol.doc. Completed SPIRIT checklist.** (DOC 121 kb)

## Abbreviations

AE: adverse events
CI: chief investigator
CoI: co-investigator
CRF: Case Report Form
DMC: Data Monitoring Committee
DRF: Doctoral Research Fellowship
ELP: erosive lichen planus
ELPV: erosive lichen planus affecting the vulva
‘hELP’: Systemic therapy for vulval Erosive Lichen Planus trial
NIHR: National Institute for Health Research
PI: principal investigator
RCT: randomised controlled trial
RN: research nurse
SF36: ‘Short Form 36’ questionnaire
TDG: Trial Development Group
TMG: Trial Management Group
TSC: Trial Steering Committee
